# Synthesis and Characterization of Some New Complexes of Magnesium (II) and Zinc (II) with the Natural Flavonoid Primuletin

**DOI:** 10.3390/molecules18077631

**Published:** 2013-07-01

**Authors:** Valentina Uivarosi, Mihaela Badea, Rodica Olar, Constantin Drǎghici, Ştefania Felicia Bǎrbuceanu

**Affiliations:** 1 Department of General and Inorganic Chemistry, Faculty of Pharmacy, Carol Davila University of Medicine and Pharmacy, 6 Traian Vuia St, Bucharest 020956, Romania; 2 Department of Inorganic Chemistry, Faculty of Chemistry, University of Bucharest, 90-92 Panduri St., Bucharest 050663, Romania; E-Mails: e_m_badea@yahoo.com (M.B.); rodica_m_olar@yahoo.com (R.O.); 3 Center of Organic Chemistry C.D. Nenitzescu, Romanian Academy, 202 B Splaiul Independenţei, Bucharest 060023, Romania; E-Mail: cst_drag@yahoo.com; 4 Department of Organic Chemistry, Faculty of Pharmacy, Carol Davila University of Medicine and Pharmacy, 6 Traian Vuia St, Bucharest 020956, Romania; E-Mail: stefaniafelicia_barbuceanu@yahoo.com

**Keywords:** 5-hydroxyflavone (primuletin), Mg(II) and Zn(II) complexes, spectral properties, thermal behavior

## Abstract

Two new metal complexes formulated as [Mg(L)_2_(H_2_O)_2_]·H_2_O (1) and [Zn(L)_2_(H_2_O)_2_]·0.5H_2_O (2), where HL = 5-hydroxyflavone (primuletin), have been synthesized and characterized by elemental and thermal analyses, molar conductance, IR, UV-Vis, ^1^H- and ^13^C-NMR, fluorescence and mass spectra. In solid state, complexes had shown higher fluorescence intensities comparing to the free ligand, and this behavior is appreciated as a consequence of the coordination process.

## 1. Introduction

**Figure 1:**
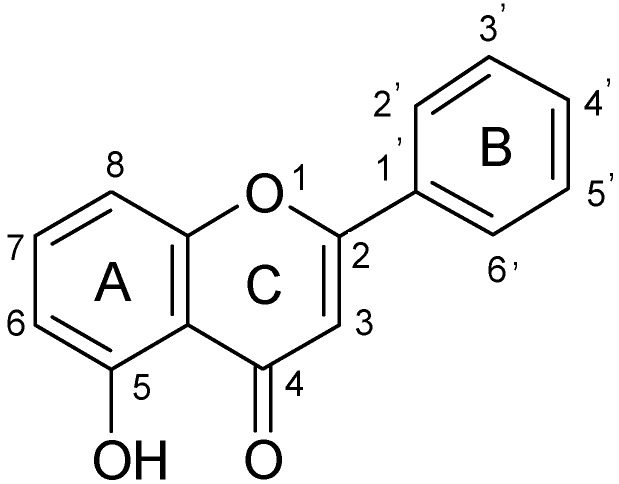
The structure of primuletin (5-hydroxyflavone).

Primuletin (5-hydroxyflavone, 5-hydroxy-2-phenyl-4*H*-1-benzopyran-4-one, Figure 1) is a naturally occurring flavone, widely distributed in plants belonging to the *Primula* and *Dionisya* species [1,2].

As for other flavonoid compounds, the antioxidant properties as well as the effects on cell lines, receptors or enzymes have attracted a major interest in order to correlate them with biological activity.

The antioxidant activity of flavonoids is generally associated with three chemical features: (i) an *ortho*-dihydroxy structure in the B-ring, (ii) the presence of a 2,3 double bond in the C-ring, and/or (iii) the presence of a 4-oxo function in the C-ring [3]. Additionally, an OH group in position 3 of the C-ring was correlated with the antioxidant properties [4]. In the case of 5-hydroxyflavone, only two of these necessary conditions are met, and consequently it has not noticeable antioxidant activity [5,6]. The monoxydroxylated primuletin has a low or absent scavenging effect [7,8] correlated with a low cytotoxicity on B 16 melanoma cells, and induced little apoptosis in human leukemia cells [9] or even had not apoptotic effect [10].

On the other side, 5-hydroxyflavone can act as activator of calcium-activate and ATP-sensitive potassium channels; the hydroxyl group in position 5 seems to be a structural requirement for a possible interaction with calcium-activated potassium channels [11]. As consequence, 5-hydroxyflavone showed full vasorelaxing effects in a comparative study involving 17 different flavones. 

From a series of 25 tested flavones, 5-hydroxyflavone proved to be had the highest androgen receptor (AR) antagonistic activity. Its action was threefold higher than that of flutamide, a well known AR antagonist used to treat prostate cancer [12].

The presence of a hydroxy group in position 5 confers to primuletin a superior activity on some enzymes comparing to unhydroxylated or methoxylated analogues. In this regard, the C(5)-hydroxyl group induce a better inhibition of phospholipase A2 group II A (PLA2–IIA) than their respective C(5)-unhydroxylated derivatives [13]. The inhibition of PLA2 leads to a decrease in eicosanoids levels, thereby reducing inflammation. The presence of OH function in C-5 position is responsible for the much higher activity of 5-hydroxyflavone comparing to 5-methoxyflavone in inhibition of nuclear factor κB (NF-κB) [14]. Since NF-κB is involved in inflammation, cell proliferation, apoptosis and angiogenesis [15], the molecules that interfere with NF-κB signaling may be useful as anti-inflammatory and anticancer agents.

Primuletin inhibits nitric oxide syntase-2 (NOS-2) induced in macrophages by lipopolysaccaride (LPS) from Escherichia coli serotype with putative antiatherogenic effect [16].

Inhibition of human cytocrome P450 1A1, 1A2, 1B1, 2C8, and 3A4 by a series of flavonoid derivatives, including 5-hydroxyflavone, was studied and structure-function relationships were established. Knowing that these enzymes are involved in the activation and detoxification of endogenous chemicals and xenobiotics, the substances that inhibit them can influence the human health. 5-hydroxyflavone is more active than flavone in inhibiting P450 1A1 and P450 1B1, less active than flavones in inhibiting of P450 1A2 and it is weak in inhibiting P450 3A4 and P450 2C9 [17].

Due to the presence of a chelating 5-hydroxy-4-keto group, 5-hydroxyflavone can act as a bidentate ligand toward metal ions. The complexation process of 5-hydroxyflavone with some metal ions was investigated mainly in solution, using spectroscopic techniques. Investigation of the solvent effects on Al(III)-5-hydroxyflavone complexes revealed that while in pure methanol a stoichimetry of 1:1 was obtained [18], in methanol/water medium at pH 6 a species with 1:2 metal:ligand stoichiometry was formed [19]. Complexes with 1:1 stoichiometry were obtained in pure methanol also for Pb(II) [20] and Zn(II) [21]. Several complexes were obtained in solid state, for example those with Co(II), Ni(II), Cu(II), V(III), and Fe(III) [22], and VO(II) [23]. A solid mixed ligands complex of Ru(II) with 5-hydroxyflavone and dimethyl sulfoxide was also reported [24]. Applications of metal complexes are mainly based on their luminiscent properties. Some examples are the use of 5-hydroxyflavone-Al(III) complex as fluorescent fluoride ion probe [25] and of [Be(5Fla)_2_] (Fla = 5-hydroxyflavonate ion) as emitting material in organic light-emitting diodes [26]. Although so far, biological applications of metal complexes of 5-hydroxyflavone have not been reported, some recent studies that evidenced the hypoglycemic activity of 3-hydroxyflavone complexes with Zn(II) [27] and VO(II) [28] are encouraging for investigating the biological activity of complexes with 5-hydroxylated analogue.

The present work adds to the efforts to obtain new complexes of 5-hydroxyflavone with potential biological activity. Two new solid compounds of Mg(II) and Zn(II) with 5-hydroxyflavone were obtained. The composition and structure of complexes were investigated by elemental and thermal analysis, IR, ^1^H- and ^13^C-NMR spectroscopy and by mass spectra analysis. The nature of complexes was determined by measuring the conductance of DMSO solutions. Fluorescence properties of complexes were determined in solid state and in solution of various solvents, comparing to those of the free ligand.

## 2. Results and Discussion

**Scheme 1:**
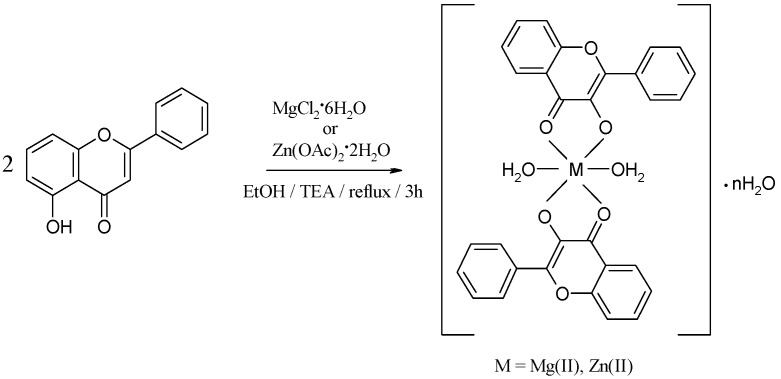
The reaction scheme for the synthesis of the complexes.

The yellow compounds obtained according to the general reaction depicted in Scheme 1 are hardly soluble in water, their solubility being presented in Table 1. Table 2 shows the analytical and molar conductance data for the complexes. The low values of the molar conductance suggest the non-electrolytic nature of the complexes.

**Table 1:** Solubility in different solvents of the parent ligand and its complexes.

Compound	DMSO	DMF	AcCN	CHCl_3_	MeOH	EtOH
HL	soluble	soluble	soluble	soluble	soluble at mild heating	soluble at mild heating
[Mg(L)_2_(H_2_O)_2_]·H_2_O (**1**)	soluble	slightly soluble	slightly soluble	soluble at mild heating	slightly soluble	slightly soluble
[Zn(L)_2_(H_2_O)_2_]·0.5H_2_O (**2**)	soluble	soluble	soluble	soluble	slightly soluble	slightly soluble

**Table 2:** Analytical and molar conductance data for the complexes.

Compound	Molecular formula	Molecular weight (g mol^−1^)	Anal. found (calcd.) (%)			Molar conductance Λ_M_ (Ω^−1^ cm^2^ mol^−1^)
			C	H	M	
[Mg(L)_2_(H_2_O)_2_]·H_2_O (**1**)	MgC_30_H_24_O_9_	552.82	64.95 (65.18)	4.24 (4.38)	4.10 (4.39)	2
[Zn(L)_2_(H_2_O)_2_]·0.5H_2_O (**2**)	ZnC_30_H_23_O_8.5_	584.90	61.40 (61.60)	4.12 (3.96)	10.92 (11.18)	3.5

### 2.1. IR Spectra

IR spectra of the ligand and the complexes bring evidences of coordination of Mg(II) and Zn(II) ions to the 5-hydroxyflavone. The data are summarized in Table 3.

**Table 3:** IR data (cm^−1^) for ligand and complexes.

Compound	ν(O-H)	ν(C=O)	ν(C=C)	ν(C-O) + δ(OH)	ν(C-O-C)	γ_w_(H_2_O)
HL	3,200–2,600 b, m	1,654 s; 1,615 s	1,587 s	1,357 m; 1,298 s	1,255 s	-
[Mg(L)_2_(H_2_O)_2_]·H_2_O (**1**)	3,600–2,600 b, m	1,634 s	1,583 s	1,361 m; 1,297 w	1,251 s	422 w
[Zn(L)_2_(H_2_O)_2_]·0.5H_2_O (**2**)	3,600–2,600 b, m	1,632 s	1,580 s	1,355 m; 1,297 w	1,250 s	546 w

b: broad; m: medium; s: strong; w: weak.

**Figure 2:**
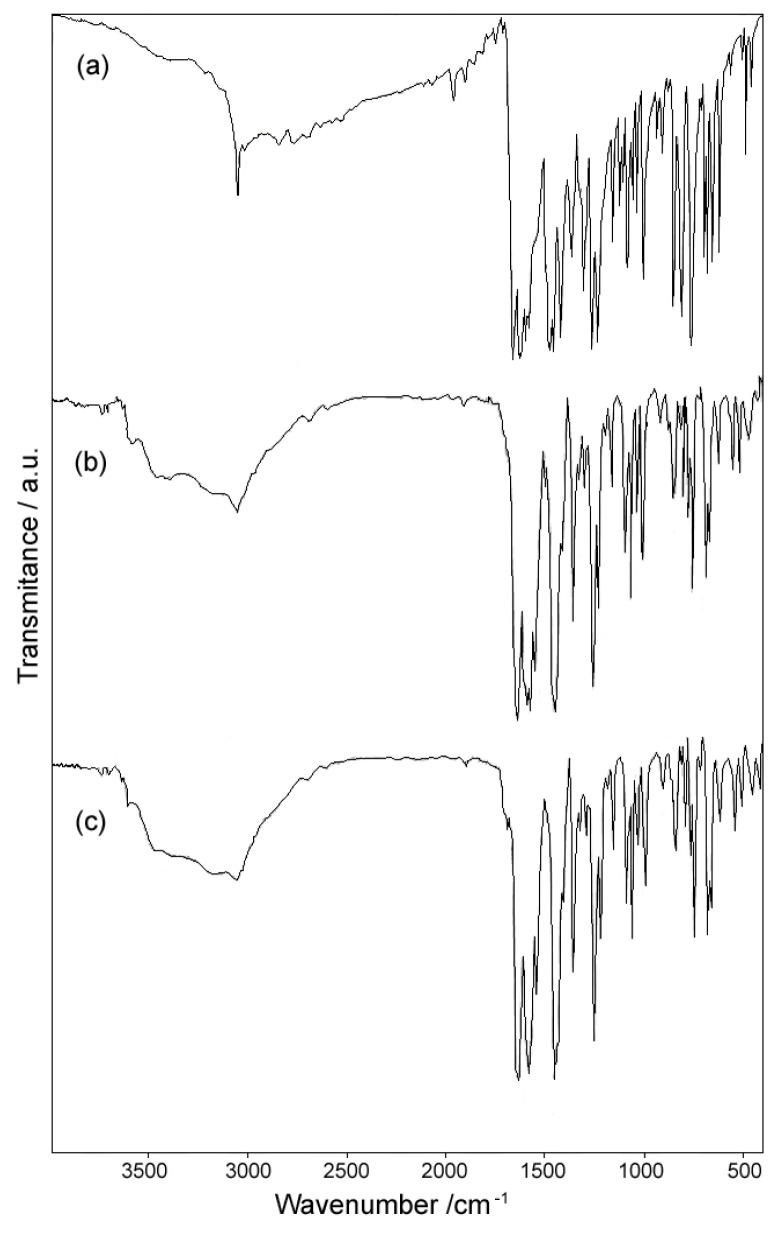
IR spectra of (**a**) 5-hydroxyflavone (HL). (**b**) [Mg(L)_2_(H_2_O)_2_]·H_2_O. (**c**) [Zn(L)_2_(H_2_O)_2_]·0.5H_2_O.

In the high wavenumber region, the IR spectrum of 5-hydroxyflavone [Figure 1a] displays an intense broad band between 2,600 and 3,200 cm^−1^. This band is due to the strong intramolecular hydrogen bond involving the OH group, a characteristic feature of 5-hydroxylated chromones [29]. A sharp and intense band present in this region at 3,059 cm^−1^ corresponds to the stretching vibration ν(C-H). In the IR spectra of the complexes (Figure 2b,c) are found a broad band between 2,600 and 3,600 cm^−1^, assigned to the presence of water molecule in the structure of the complexes. Simultaneously, the ν(C-H) band is weakened and broadened by overlapping.

In the 1,550–1,750 cm^−1^ region, the ν(C=O) vibration of 5-hydroxyflavone generates two intense bands, placed at 1,654 and 1,615 cm^−1^. In the IR spectra of the complexes, a single strong band appears at around 1,633 cm^−1^. The displacement of ~20 cm^−1^ suggests the involvement of C=O group in coordination. The strong band characteristic for ν(C=C) which appears at 1,587 cm^−1^ in the IR spectrum of the ligand, is slightly shifted in the IR spectra of complexes, supporting that this bond is unaffected by coordination.

Between 1,000 and 1,500 cm^−1^ in the IR spectra bands associated with δ(OH) mode are presented, mixed with ν(C=O), ν(CC) and aromatic ring deformation. The strong band at 1,298 cm^−1^ from the coupled vibration ν(C-O) + δ(OH) [30] in the IR spectrum of 5-hydroxyflavone, appears very weakened in the IR spectra of complexes, that suggests its involvement in coordination in the deprotonated form. The ν(C-O-C) frequency is not shifted in the IR spectra of complexes comparing to the IR spectrum of the ligand, indicating that the ring oxygen is not involved in coordination.

The presence of the coordinated water in the structure of the complexes is indicated by the wagging frequencies at 422 for complex **1** and 546 cm^−1^ for complex **2**, respectively [31].

### 2.2. UV-Vis Spectra

The diffuse reflectance electronic spectra of 5-hydroxyflavone exhibit two intensive absorption bands originated from π–π* transitions; the band centred at 396 nm (band I) is due to the transition localized within the B ring of cinnamoyl system, whereas the one centred at 280 nm (band II) may be assigned to transitions in the ring A of benzoyl system [32]. UV-Vis data for ligand and complexes are presented in Table 4.

**Figure 3:**
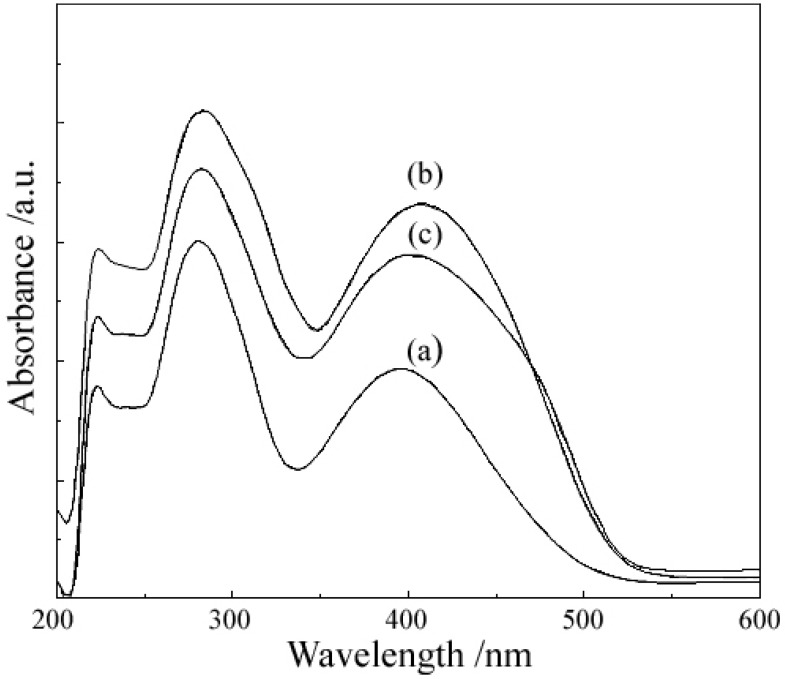
Diffuse reflectance electronic spectra of (**a**) 5-hydroxyflavone (HL). (**b**) [Mg(L)_2_(H_2_O)_2_]·H_2_O. (**c**) [Zn(L)_2_(H_2_O)_2_]·0.5H_2_O.

The characteristic features in the spectra of complexs are the bathochromic shifts of the two bands of the ligand, due to the extension of the conjugated system with the complexation, as can be shown in Figure 3.

**Table 4:** UV-Vis data for ligand and complexes.

Compound	λ_max_ (nm)	
	Band I	Band II
HL	396	280
[Mg(L)_2_(H_2_O)_2_]·H_2_O (**1**)	406.5	284
[Zn(L)_2_(H_2_O)_2_]·0.5H_2_O (**2**)	403.5	283

### 2.3. ^1^H-NMR and ^13^CNMR Spectra

Complexation of Mg(II) and Zn(II) to 5-hydroxyflavone was investigated by ^1^H- and ^13^C-NMR, using DMSO-d_6_ as solvent. The ^1^H chemical shifts for ligand and complexes are presented in Table 5 and were assigned based on the literature data [33], while the ^13^C- chemical shifts and their assignment [34] are done in Table 6. The main difference observed in the ^1^H-NMR spectrum of complexes is the absence of chemical signal of hydrogen from 5-OH phenolic group. Some of the other proton signals are shifted to lower frequencies relative to the free ligand or are broadened due to the fact that coordination increases the planarity of flavonoid molecule, therefore decreasing the mobility of the protons [35]. Slightly shifts of the chemical signals of carbon atoms belonging to the ring A and C are observed in the ^13^C-NMR spectra of complexes comparing to that of the free ligand. Instead, the signals of carbon atoms from the side ring B are practically unchanged, and these observation support the involvement of the chemical groups (phenolic OH and carbonyl C=O) on the rings A and C in complexation.

**Table 5:** The ^1^H chemical shifts (ppm) for ligand and complexes.

Compound	δ of ^1^H (*J,* Hz)									
	H-3	H-6	H-7	H-8	H-3′	H-4′	H-5′	H-2′	H-6′	OH
HL	7.1 (s)	6.8 (d, 8.3)	7.6 (t, 8.1)	7.2 (d, 8.1)	7.6 (m)			8.1 (dd, 7.8, 1.7)		12.55 (s)
(**1**)	6.8 (s)	6.4(bd, 9.9)	7.3 (t, 8.2)	6.3 (bd, 8.2)	7.6 (m)			8.0 (dd, 8.0, 2.6)		-
(**2**)	7.0 (s)	6.5 (d, 8.4)	7.4 (t, 8.4)	6.7 (d, 8.4)	7.6 (m)			8.1 (dd, 8.2, 1.7)		-

**Table 6:** The ^13^C chemical shifts (ppm) for ligand and complexes.

Compound	δ of ^13^C								
	C-2	C-3	C-4	C-5	C-6	C-7	C-8	C-9	C-10
HL	164.1	105.7	183.2	159.8	111.0	135.9	107.5	155.9	110.1
(**1**)	171.4	106.2	180.9	161.1	116.9	134.9	96.8	158.2	113.4
(**2**)	171.4	106.0	183.1	162.1	117.1	135.4	99.7	157.78	112.2
**Compound**	**δ of ^13^C**								
	**C-1′**	**C-2′**	**C-6′**	**C-3′**	**C-5′**	**C-4′**			
HL	130.53	126.6		129.2		132.3			
(**1**)	131.11	126.1		129.2		131.5			
(**2**)	130.72	126.3		129.2		131.9			

### 2.4. Mass Spectra

**Figure 4:**
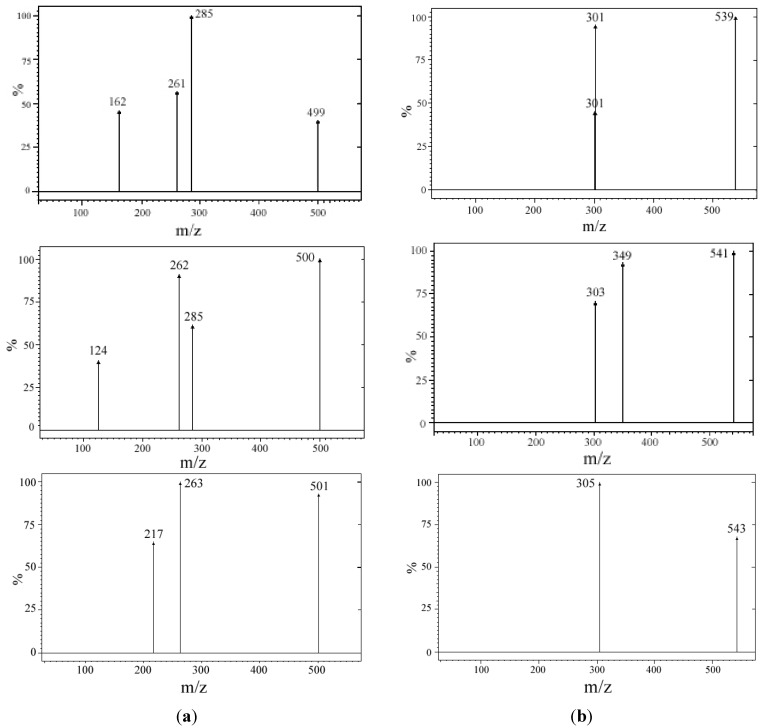
Mass spectra of (**a**) [Mg(L)_2_(H_2_O)_2_]·H_2_O. (**b**) [Zn(L)_2_(H_2_O)_2_]·0.5H_2_O.

The direct injection of a acetonitrile solution of the complex into a ESI interface leads to the protonated molecular ion of the ligand [M+H]^+^ *m/z* = 239, obtained as main ion. However, if a solution of complex in acetonitrile/water with 0.1% ammonia 9/1 in the +ESI-MS spectrum, the protonated molecular ion of the complex could be also observed (Figure 4). The fragments resulted by collision with argon at a pressure of 1.5 mTorr comply the natural isotopic abundances of the metallic elements (Table 7). The data from the mass spectra suggest the 1:2 molar ratio metal ion:ligand.

**Table 7:** Nominal *m/z* of the ions observed in the positive ion mode ESI-MS fingerprint of the complexes and the identified structure.

ML_n_ species	Protonated molecular ion [ML_n_+H]^+^ (*m/z*)	Fragment at 5 eV and 1.5 mTorr argon (*m/z*)	Type of fragment	Fragmentation
^24^MgL_2_	499	261	^24^MgL^+^	[^24^MgL+H-L]^+^
^25^MgL_2_	500	262	^25^MgL^+^	[^25^MgL+H-L]^+^
^26^MgL_2_	501	263	^26^MgL^+^	[^26^MgL+H-L]^+^
^64^ZnL_2_	539	301	^64^ZnL^+^	[^64^ZnL+H-L]^+^
^66^ZnL_2_	541	303	^66^ZnL^+^	[^66^ZnL+H-L]^+^
^68^ZnL_2_	543	305	^68^ZnL^+^	[^68^ZnL+H-L]^+^

### 2.5. Thermal Behavior

The results regarding the thermal decomposition of complexes are described in the following section and are summarized in the Table 8.

**Table 8:** Thermal degradation data (in synthetic air) for the complexes.

Complex	Step	Thermal effect	Temperature range/°C	Δm_exp_/%	Δm_calc_/%	Chemical process
[Mg(L)_2_(H_2_O)_2_]·H_2_O (**1**)	1.	Endothermic	52–90	3.0	3.3	H_2_O loss
	2.	Endothermic	120–160	3.1	3.3	H_2_O loss
	3.	Endothermic	170–206	3.2	3.3	H_2_O loss
	4.	Exothermic	408–900	56.3		Partial oxidative degradation of organic part
	Residue (MgO + organic residue)			34.4		
[Zn(L)_2_(H_2_O)_2_]·0.5H_2_O (**2**)	1.	Endothermic	54–75	1.7	1.6	0.5 H_2_O loss
	2.	Endothermic	85–128	6.1	6.2	2H_2_O loss
	3.	Exothermic	300–750	78.5	78.3	Oxidative degradation of organic part
	Residue (ZnO)			13.7	13.9	

#### 2.5.1. Thermal Decomposition of [Mg(L)_2_(H_2_O)_2_]·H_2_O

**Figure 5:**
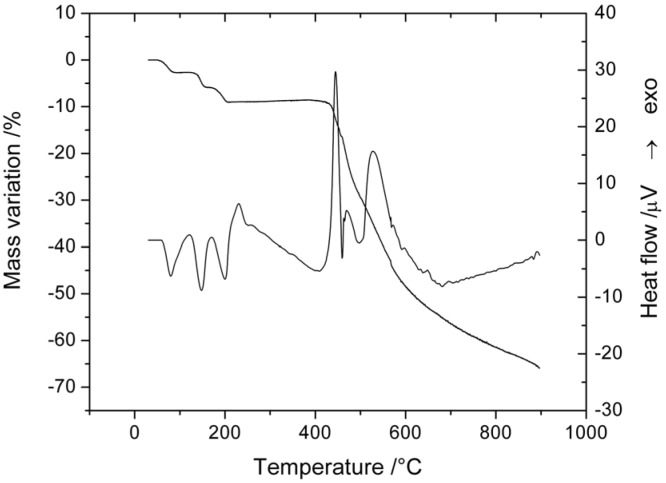
TG and DTA curves of [Mg(L)_2_(H_2_O)_2_]·H_2_O.

Thermal decomposition of complex **1** undergoes in four well-defined steps (Figure 5, Table 8). The first step corresponds to the loss of one water molecule. This endothermic step occurs at low temperature confirming the lattice nature of these. The next two endothermic events, could be associated with the release of the two coordinated water molecules.

The anhydrous compound is stable over a large temperature range (206–408 °C). The last exothermic decomposition step corresponds to oxidative degradation of the organic part. Tacking into accord the aspect of TG curve and the value of mass loss we can conclude that the oxidative degradation of organic part is not finished at 900 °C.

#### 2.5.2. Thermal Decomposition of [Zn(L)_2_(H_2_O)_2_]·0.5H_2_O

**Figure 6:**
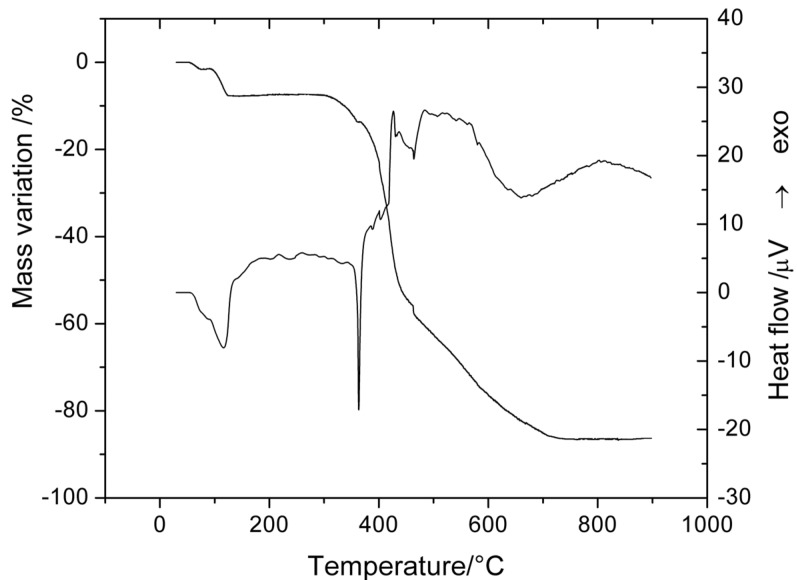
TG and DTA curves of [Zn(L)_2_(H_2_O)_2_]·0.5H_2_O.

The analysis of TG and DTA curves (Figure 6) allowed us to establish the final formula of this compound Thus, the first endothermic mass loss in the 50–90 °C temperature range corresponds to the lattice water molecules release. The two coordinated water molecules are loss in a single endothermic step. The anhydrous complex is stable up to 300 °C when it starts the oxidative degradation of organic ligand. This exothermic step is a complex one, consisting in at least three events, as shown both TG and DTA curves. The final residue is zinc oxide as powder X-ray diffraction indicated.

### 2.6. Fluorescent Properties

**Figure 7:**
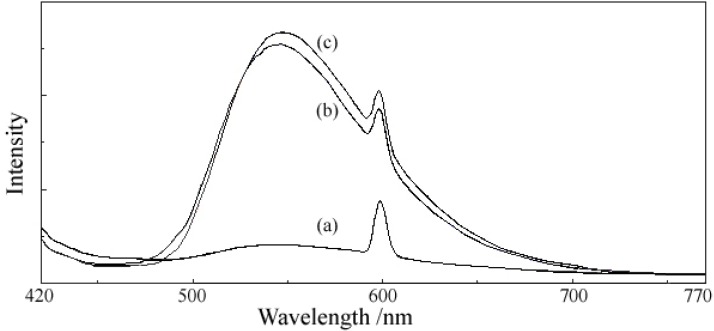
The emission spectra at λ_exc_ = 400 nm of (**a**) 5-hydroxyflavone (HL). (**b**) [Mg(L)_2_(H_2_O)_2_]·H_2_O. (**c**) [Zn(L)_2_(H_2_O)_2_]·0.5H_2_O.

**Figure 8:**
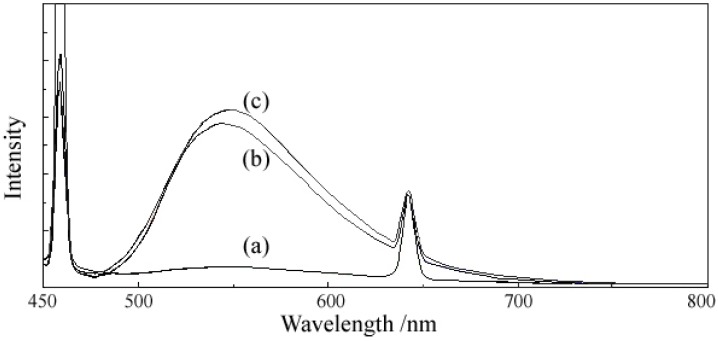
The emission spectra at λ_exc_ = 429 nm of (**a**) 5-hydroxyflavone (HL). (**b**) [Mg(L)_2_(H_2_O)_2_]·H_2_O. (**c**) [Zn(L)_2_(H_2_O)_2_]·0.5H_2_O.

The fluorescence emission spectra (Figure 7 and Figure 8) were recorded at two excitation wavelengths, 400 and 429 nm, respectively, both for ligand and complexes. The results presented in Table 9 let us draw the following conclusions: (i) 5-hydroxyflavone itself exhibits a strong fluorescence; (ii) at the excitation wavelength of 400 nm (an absorption maximum in the UV-Vis spectra of ligand and complexes), the fluorescence emission intensity of complexes was markedly higher than that of the ligand at ~600 nm, and a new band of very high intensity appeared at ~545 nm in the fluorescence emission spectra of complexes; (iii) at the excitation wavelength of 429 nm (descending branch of the peak in the UV-Vis spectrum), the fluorescence emission intensity of complexes was a little bit higher than that of the ligand at 642 nm, the new band of very high intensity appeared at ~ 548 nm in the fluorescence spectra of complexes was still present, while the fluorescence emission intensity of complexes was smaller than that of the ligand at 458 nm. The enhancement in the fluorescence intensity of complexes may be related to the formation of a chelate ring via coordination of the metal ion, which increases the rigidity of the ligand structure and enhances the fluorescence quantum yield by reducing the probability of non-radiative dissipation process. The new emission band that appeared in the emission spectra of complexes could be appreciated as a evidence for the formation of a new bond, a metal-ligand bond.

**Table 9:** Fluorescence data for ligand and complexes.

Compound	Excitation wavelength λ_exc_ = 400 nm		Excitation wavelength λ_exc_ = 429 nm	
	Emission wavelength λ_em_ (nm)	Relative fluorescence intensity (a.u.)	Emission wavelength λ_em_ (nm)	Relative fluorescence intensity (a.u.)
HL	540 599	82.43 174.87	456 545 642	>1000 73.23 323.29
[Mg(L)_2_(H_2_O)_2_]·H_2_O (**1**)	545 598	510.61 379.94	459 548 642	824.65 576.89 331.28
[Zn(L)_2_(H_2_O)_2_]·0.5H_2_O (**2**)	548 598	536.83 410	458 548 642	727.71 627.11 346.30

## 3. Experimental

All reagents and solvents were of analytical reagent grade and were used without further purification. 5-hydroxyflavone, MgCl_2_·6H_2_O and Zn(OAc)_2_·2H_2_O were purchased from Aldrich Chemical Co., Schnelldorf, Germany.

Elemental analyses were performed using a Perkin Elmer PE 2400 analyser (for C, H, N, S) and a Shimadzu AA 6300 spectrometer (for magnesium and zinc). The conductivity was measured with a Consort C830 (Turnhout, Belgium) conductimeter with an SK10T platinum electrode embedded in glass (cell constant 1.0 cm^−1^). IR spectra were recorded using KBr pellets on a FT-IR VERTEX 70 (Bruker) spectrometer in the range 400–4,000 cm^−1^. Electronic spectra by diffuse reflectance technique, with magnesium oxide as reference sample, were recorded in the range 200–600 nm, on a Jasco V 650 spectrophotometer. Fluorescence spectra were recorded on a Jasco FP 6500 spectrofluorometer. For UV-Vis and fluorescence measurements, the solid sample was diluted with magnesium oxide (~1 mg of solid sample in 50 mg MgO). The ^1^H and ^13^C-NMR spectra were recorded on a NMR Varian Gemini 300 BB spectrometer working at 300 MHz for ^1^H and 75 MHz for ^13^C in in DMSO-d_6_. All chemical shifts are reported in δ (ppm) using TMS as the internal standard. Mass spectra were recorded by electrospray ionization tandem mass spectrometry (ESI-MS) technique. Solutions of 5 mg/mL in acetonitrile/water with 0.1% ammonia in 9/1 (v/v) ratio were injected directly into the electrospray interface of a 1200 L/MS/MS (Varian) mass spectrometer using a Prostar 240 SDM (Varian) pump. Molecular ions scanning range (m/z) was 150–1,500. The heating curves (TG, and DTA) were recorded using a Labsys 1200 SETARAM thermobalance with a sample weight between 10–14 mg over the temperature range of 20–900 °C and a heating rate of 10 °C min^−1^. The measurements were carried out in synthetic air atmosphere (flow rate 16.66 mL min^−1^), using alumina crucible. The X-ray powder diffraction patterns were collected on a DRON-3 diffractometer with a nickel filtered Cu K_α_ radiation (λ = 1.5418 Å) in 2θ range of 5–70°, a step width of 0.05° and an acquisition time of 2 s per step.

Synthesis of complexes was carried out following the general procedure described in [36]: an ethanolic solution (25 mL) of 5-hydroxyflavone (2 mmol, 0.476 g), deprotonated with TEA (2 mmol, 0.28 mL) was added to an ethanolic solution (5 mL) of metal salt (1 mmol, 0.2033 g MgCl_2_·6H_2_O or 0.2195 g Zn(OAc)_2_·2H_2_O). The reaction mixture was refluxed for 3 h. The yellow product formed was filtered off, washed several times with small amounts of ethanol, and dried in air (Yield: 92.8% [Mg(L)_2_(H_2_O)_2_]·H_2_O, 81% [Zn(L)_2_(H_2_O)_2_]·0.5H_2_O). 

## 4. Conclusions

Primuletin (5-hydroxyflavone) forms in the selected working conditions ions two new complexes with Mg(II) and Zn(II), which have been characterized by elemental analyses, thermal analysis (TG, DTA), conductometric measurements and several spectroscopic techniques (IR, UV-Vis, ^1^H- and ^13^C-NMR, mass spectra). From the experimental data, the composition and structure, as well as the non-electrolytic nature of complexes have been established. The obtained complexes possess strong fluorescent properties when excited at 400 or 429 nm, valuable for future applications of these complexes. The biological potential of the complexes also worth explored.
